# Family Related Variables’ Influences on Adolescents’ Health Based on Health Behaviour in School-Aged Children Database, an AI-Assisted Scoping Review, and Narrative Synthesis

**DOI:** 10.3389/fpsyg.2022.871795

**Published:** 2022-08-10

**Authors:** Yi Huang, Michaela Procházková, Jinjin Lu, Abanoub Riad, Petr Macek

**Affiliations:** ^1^Institute for Research of Children, Youth and Family, Faculty of Social Studies, Masaryk University, Brno, Czechia; ^2^Department of Psychology, Faculty of Social Studies, Masaryk University, Brno, Czechia; ^3^AoFE, Xi’an Jiaotong-Liverpool University, Suzhou, China; ^4^Czech National Centre for Evidence-Based Healthcare and Knowledge Translation, Department of Public Health, Faculty of Medicine, Masaryk University, Brno, Czechia

**Keywords:** adolescents’ health, HBSC database, AI-assisted scoping review, family environment, parenting behaviour

## Abstract

**Objects:**

Health Behaviours in School-aged Children (HBSC) is an international survey programme aiming to investigate adolescents’ health behaviours, subjective perception of health status, wellbeing, and the related contextual information. Our scoping review aimed to synthesise the evidence from HBSC about the relationship between family environmental contributors and adolescents’ health-related outcomes.

**Methods:**

We searched previous studies from six electronic databases. Two researchers identified the qualified publications independently by abstract and full-text screening with the assistance of an NLP-based AI instrument, ASReview. Publications were included if they were based on HBSC data and investigated the effects of family environment on adolescents’ health outcomes. Researches addressed family-related factors as mediators or moderators were also included.

**Results:**

A total of 241 articles were included. Family environmental contributors could be mapped into six categories: (1) Demographic backgrounds (*N* = 177); (2) General family’s psycho-socio functions (*N* = 44); (3) Parenting behaviours (*N* = 100); (4) Parental health behaviours (*N* = 7); (5) Family activities (*N* = 24); and (6) Siblings (*N* = 7). Except for 75 papers that assessed family variables as moderators (*N* = 70) and mediators (*N* = 7), the others suggested family environment was an independent variable. Only five studies employed the data-driven approach.

**Conclusion:**

Our results suggest most research studies focussed on the influences of family demographic backgrounds on adolescents’ health. The researches related to parental health behaviours and siblings are most inadequate. Besides, we recommend further research studies to focus on the mediator/moderator roles of the family, for exploring the deep mechanism of the family’s impacts. Also, it would be valuable to consider data-driven analysis more in the future, as HBSC has mass variables and data.

## Introduction

Adolescence is a vulnerable developmental stage because of the significant changes in hormone levels, which may affect psychological characteristics (Call et al., 2002). Thus, adolescents tend to experience more subjective health complaints than before (Potrebny et al., 2019) and report lower life satisfaction (Inchley et al., 2020). Moreover, they face a greater risk of developing risky health behaviours, such as substance use, problematic Internet use, and others (Whitesell et al., 2013; Chassin et al., 2014; Shek and Yu, 2016).

It has been known that family influences the health-related outcomes of youth. According to ecological theory, children directly interact with three environments: family, school, and peers. They play a significant role in children’s physical and psychological development (Bronfenbrenner, 1979). Ample empirical evidence has shown that family environment significantly affects adolescents’ health. For instance, in the United States, a family’s disadvantaged socioeconomic status (SES) is linked to children’s overweight because such households provide opportunities for sedentary behaviours and inadequate physical activities, which increase children’s risks of obesity (Tandon et al., 2012). Furthermore, an American national survey suggested that family structure is associated with children’s physical and mental health (Bramlett and Blumberg, 2007). In detail, researchers found that compared to children living with two biological parents, children in single-mother or grandparent-only families have poorer health outcomes, for example, higher risk of asthma-related problems, cognitive disorders, and affective difficulties.

Except for the family demographic factors, parenting and parental health-related behaviours play an important role in children’s physical health and health-related behaviours. A meta-analysis work pointed out that authoritative parenting style is linked to lower and authoritarian parenting style is linked to higher children’s and adolescents’ externalising disorders (Pinquart, 2017). A previous study also noted that the oral health behaviour of parents predicts their children’s oral health status (Bozorgmehr et al., 2013). And, parental tobacco usage is associated with adolescents’ smoking behaviours (Hublet et al., 2007).

### The Health Behaviour in School-Aged Children Study

To investigate adolescents’ health-related issues, World Health Organization launched a collaborative cross-national project named Health Behaviour in School-Aged Children (HBSC), which is based on a series of standard self-reported student questionnaires. HBSC targets students in 11/13/15-year-old grade. The first HBSC survey was administered in 1983–1984 in five countries, and since 1985, the surveys have been conducted every 4 years (HBSC, n.d.). The latest survey data was collected in 2017–2018 in fifty countries and regions (considered as countries, including England, Scotland, Wales, Belgium Flemish, and Belgium French) in Europe, North America, Israel, Turkey, Kyrgyzstan, and Uzbekistan (Inchley et al., 2020). To obtain a nationally representative sample, the data collection should be conducted at the national level in each country/region. The sampling design was based on a two-stage clustered hierarchical approach. In the first stage, the basic unit is school class or school when the class lists are not accessible (UNICEF-IRC, n.d.). The HBSC protocol for each country or region contains three types of questions: mandatory HBSC items that all members of the HBSC project should include in the survey, optional HBSC items that a country can choose to administer or omit from the questionnaire, and national items that a country can design based on its background and include in the survey.

The key outcome variables of HBSC are youth’s health-related behaviours, subjective perception of health, and wellbeing. The initial design of the HBSC project was based on Bronfenbrenner’s ecological theory, which aimed to investigate adolescents’ health issues from an environmental perspective, ranging from microsystem (including family, school, and peer relationships), mesosystem representing the interactions within microsystem, exosystem (e.g., community), to macrosystem (e.g., country-level background) (Aarø et al., 1986). According to the newest protocol of HBSC project, except for the ecological framework, HBSC currently integrates additional three conceptual approaches to collect adolescents’ social contextual information (Inchley et al., 2018): social psychological perspective, public health or epidemiological approach, and developmental or biological viewpoint. The social psychological perspective addresses the influences of social psychological factors at individual level, such as perceived social supports and social strains. The public health perspective mainly focuses on the adolescent group at risks and the identified risk factors behind, as well as the trend of the risks. The biological conceptual framework considers the puberty maturation stage’s effect on adolescents’ wellbeing, health, and health behaviours. Under the integration of four types of conceptual frameworks, HBSC helps us gain new insight into the social determinants of adolescents’ health and wellbeing (World Health Organization [WHO], 2021).

### The Current Study

Many studies using the HBSC database have noted that family environment influences adolescents’ health-related outcomes, including physiological and psychological health, wellbeing, and health-protective and risk behaviours. Regarding the family environment information, since 2013 HBSC has included three sections: family culture, social inequality, and migration (Currie et al., 2014; Inchley et al., 2018). In the family culture section, HBSC 2017/2018 projects mandatorily comprised the following variables: family structure, ease of family communication, and family support measured by the family subscale of the Multidimensional Scale of Perceived Social Support. Social inequality section essentially investigated parental employment status, and family affluence measured by Family Affluence Scale (FAS), which was a series of items about material assets and activities. Migration section must asked the participants about the born country information of themselves and their parents.

However, to our best knowledge, few studies have systematically synthesised the effects of the family on various outcomes from HBSC. This scoping review aimed to identify and summarise the family effects from existing HBSC based studies, focussing on synthesising the categories of family variables that may affect children’s health-related outcomes. This review study aspired to provide the background for HBSC related studies, and it would guide further HBSC related research focussing on family environmental and other social contextual factors.

## Methods

This scoping review work was not aiming for interventional research; thus, we followed the scoping review guidelines provided by JBI Manual for Evidence Synthesis (Aromataris and Munn, 2020). This scoping review project’s protocol was first registered with the Open Science Framework on April 1, 2021. All materials, including the original protocol, revised protocol, and database search results, are accessible through https://doi.org/10.17605/OSF.IO/JY29A.

### Search Strategy

According to the JBI guidelines, the search strategy should be formulated according to three basic items: population, concept, and context. In our research, the target population was adolescents. As discussed, the HBSC project investigates the background, health behaviours, and psychological or physical wellbeing of 11 to 15 years old adolescents. Therefore, the context of HBSC is already limited in terms of the population and one of the concepts of this scoping review, health. The other key concept of the current review was the family environment. Thus, for the literature search, we adopted the following Boolean phase: (famil* OR parent* OR caregiv*) AND (HBSC OR “Health Behaviour in School-aged Children”). The selected electronic databases were APA PsycInfo, APA PsycArticles, Scopus, Web of Science, PsyArxiv, and MedRxiv. As no HBSC-based systematic reviews or systematic scoping reviews on the relationship between family and children’s health outcomes were published or registered before, we did not set the time limit when searching. The search strategies were consulted and confirmed with a specialist in the systematic review field from Czech Evidence-Based Healthcare JBI Centre of Excellence & Cochrane Czech Republic of Czech National Centre for Evidence-Based Healthcare and Knowledge Translation (personal communication on 07/04/2021 and 12/04/2021), and the final search strategy was executed on April 12, 2021.

### Inclusion and Exclusion Criteria

The inclusion criteria of this scoping review specified that included investigations should (1) be original studies based on HBSC; (2) consider the family environment as one of the contextual contributors to adolescents’ health outcomes; and (3) be published in the English language. (1) Non-original empirical research, such as literature reviews and letters to the editor; (2) studies that were not based on the HBSC database but only adopted part of HBSC scales; and (3) publications not in English were excluded.

### Screening

We employed Endnote X9 as the references manager to organise the search results and remove duplicates. Two stages of screening were conducted.

In the first stage, screening was based on titles and abstracts. Two coders completed the abstract screening independently with an AI-assistant tool, ASReview, which adopted Natural Language Processing technique to promote screening efficiency (van de Schoot et al., 2021). In the beginning, twenty-five abstracts were randomly selected to train the two coders. A coder could end the screening if ASReview yielded five continuous irrelevant abstracts. The consistency of the two coders’ decisions had to exceed 75% (Aromataris and Munn, 2020). Another batch of 25 abstracts for training purposes was considered if the first consistency were not satisfied. In the current study, the decision consistency between two coders across the first twenty-five abstracts was 80%. After solving the disagreement about inclusion criteria through discussion, two researchers coded another random 25 publications and reached 84% consistency. The formal abstract screening cut-off criterion was set as continuous twenty irrelevant studies found by ASReview. The screening consistency at this stage was 88.19%.

The second stage focussed on the full-text screening. Like the abstract screening, according to JBI Manual for Evidence Synthesis, a pilot training procedure was recommended before the formal full-text screening (Aromataris and Munn, 2020). Inclusive/exclusive decisions in the pilot stage of two coders were compared, and 90% agreement was achieved, suggesting that formal full-text screening could be conducted. The final consistency between the two coders was 92.5%. All the disagreements were settled through a discussion.

### Data Extraction and Synthesis

For included trials, one researcher extracted data, and another researcher managed the confirmation of the extractions. Final data extraction was completed in November 2021. Information extracted from eligible publications included author(s), year of publication, the country where the data was from; continent area(s); sample size; family-related variable(s), the measurement(s) of family variable(s), adolescents’ health outcome(s), the measurement(s) of health outcome(s), the role of family variable(s), and the design used to investigate the relationship between family and youth’s health. Based on the extracted information, we synthesised the categories of family contributions.

## Results

### Descriptive Statistics

**FIGURE 1:**
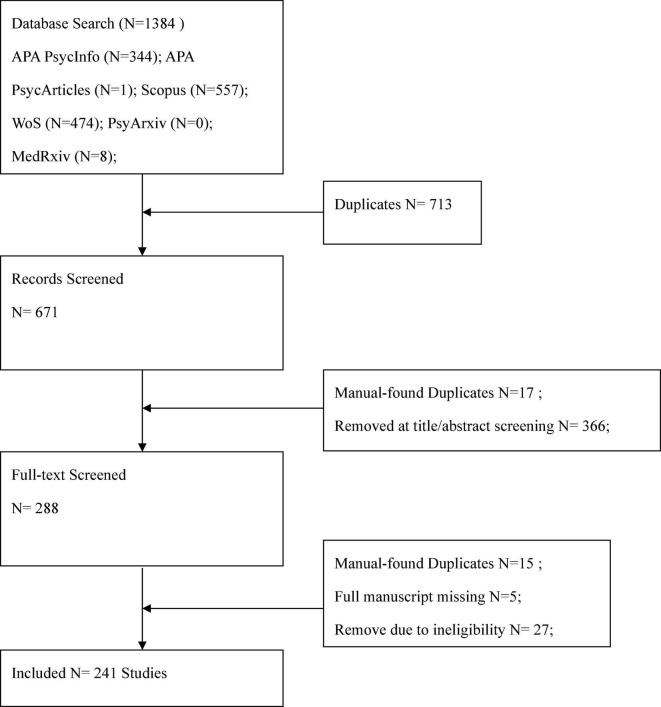
Flow chart illustrating the steps of data collection.

Overall, 634 manuscripts were retrieved, and 241 (38.01%) were included in the review. The flow diagram of the selection process is depicted in Figure 1.

Most studies (*N* = 29, 12%) were published in 2020. Only one study was published before 2000. Overall, 177 studies were conducted in European regions, 20 in North America, and 7 in the Middle East. Nineteen articles combined the European and North American data, and 18 studies were global.

### Synthesis

The current research organised family environmental factors into six thematic categories: (1) demographic backgrounds, (2) general family’s psychosocial functions, (3) parenting behaviours, (4) parental health behaviours, (5) family activities, and (6) siblings.

***Demographic backgrounds (see the Supplementary Material “data extraction_T1_back”)***

Our scoping review suggested that most studies (*N* = 177, 73.44%) (see the Supplementary Material) provided evidence supporting the effects of family-related demographic background on youth’s health.

Family socioeconomic status (SES) was the most involved demographic factor (*N* = 160). Even though SES is a controversial concept belonging to “social inequality” section in HBSC protocol, it is still plausible to categorise SES as a demographic variable, because it reflects the individual-level social inequality, instead of national-level social inequality (Ottova et al., 2012). Also, suggested by other studies, we entered the SES as a demographic factor (Vereecken et al., 2009; De Clercq et al., 2014).

Socioeconomic status is a complicated concept investigated from diverse perspectives. One hundred eleven articles (see the Supplementary Material “data extraction_T1_SES_FAS”) adopted the Family Affluence Scale (FAS) measurement to describe family SES. Four articles among them computed the Yitzhaki index based on FAS scores to describe the relative family SES (Elgar et al., 2013, 2016, 2018; Sentenac et al., 2017). Additionally, the parental occupational social class level was used as an index of SES in 27 research studies (see the Supplementary Material “data extraction_T1_SES_OSC”). Moreover, parental educational level was used to assess the SES level in ten articles while adolescents’ personal educational track was utilised to depict family SES in four studies (see the Supplementary Material “data extraction_T1_SES_Edu”). Twenty-nine (18.01%) studies investigated the relationship between SES and adolescents’ health outcomes across time waves.

Sixty-nine studies noted the association between family structure and adolescents’ health outcomes. Most family structure-related studies (*N* = 67) targetted the household composition, which referred to the composition of family members living in the same home with the adolescents (see the Supplementary Material “data extraction_T1_HouseCom”). Only two papers investigated the relationship between household composition and adolescents’ health outcomes over time (Levin et al., 2012b; Sumskas et al., 2012). Other studies adopted the cross-sectional design to investigate this link. Except for household composition, four studies also considered the effect of the number of siblings (Levin and Currie, 2010; de Grado et al., 2018; Rouche et al., 2019; Fismen et al., 2020).

Eleven studies examined the link between immigrant status and adolescents’ health. All studies used a cross-sectional design. One study was done in the middle-east region (Walsh et al., 2018), and the remaining ten studies that have considered immigrant background were conducted in Europe. No research has explored changes in the effects of immigrant background over time.

***General family’s psychosocial functions (see the Supplementary Material “data extraction_T2_ GeneralPsysocio”)***

The association between adolescents’ health outcomes and family’s general psychosocial functions, including family communication, satisfaction with family relationships, and family support, was assessed in 44 studies. There was only one longitudinal investigation to assess such a link.

Most research studies on the relationship between family communication and adolescents’ health outcomes (*N* = 12) have focussed on family communication’s influence on health behaviours, including substance use (Kuntsche and Kuendig, 2006; Zaborskis and Sirvyte, 2015; Sumskas and Zaborskis, 2017; Moore et al., 2018; dos Santos et al., 2020), food habits (Moore et al., 2018; dos Santos et al., 2020), and physical activity and sedentary behaviour (dos Santos et al., 2020). Six assessed the mental health outcome(s), involving internalising/externalising problems (Paclikova et al., 2019), subject wellbeing (Moore et al., 2018), emotional symptoms (Molcho et al., 2007; Moore et al., 2018), suicide and self-harm (Zaborskis et al., 2016), loneliness (Favotto et al., 2019), and positive attitude (Malinowska-Cieślik et al., 2019). Only one research incorporated global health (subjective psychosomatic health complaints) as an outcome (Tabak and Mazur, 2016). Another study explored the moderating effect of family communication, which demonstrated that family communication modified the relationship between computer-mediated communication and loneliness (Favotto et al., 2019).

Analogously, twelve studies employed a cross-sectional design to examine the effects of the satisfaction with family relationships on adolescents’ health. Seven of them considered health behaviours as outcomes, including substance use (Zaborskis and Sirvyte, 2015; García-Moya et al., 2017; Sumskas and Zaborskis, 2017; Lee et al., 2020), physical activity (Veloso et al., 2012; Urchaga et al., 2020), and toothbrushing (Levin and Currie, 2010). Five studies investigated the effect on adolescents’ mental health, such as suicide and self-harm (Samm et al., 2010; Zaborskis et al., 2016), life satisfaction (Resnick, 2014; Moreno-Maldonado et al., 2020a; Urchaga et al., 2020), emotional wellbeing (Samm et al., 2010; Resnick, 2014; Urchaga et al., 2020). One article addressed life satisfaction, health-related quality of life, self-reported health, and psychosomatic complaints as a global health outcome (Moreno-Maldonado et al., 2020b). Two studies analysed the moderating role of satisfaction with family relationships. In detail, an article suggested that such satisfaction can interact with adolescents’ emotional control and peers’ conventional behaviours to influence their frequency of drunkenness and tobacco use, respectively (Garcia-Moya et al., 2017). Another article examined whether family environmental contributors can decrease the negative influences of bullying experiences on adolescents’ mental health through family relationships satisfaction, parental support, family resources, and parent-child communications, although the findings indicated no significant moderating effect of all the family factors (Resnick, 2014).

Twenty-five studies investigated the relationship between family support and adolescents’ health. The majority reported the family support’s influence on adolescents’ mental health (*N* = 13, 52%). The remaining articles measured other health outcomes, including health behaviours (*N* = 8), sleep quality as a physical health aspect (*N* = 1), and global health conditions (*N* = 4). Two studies suggested family support moderating the relationship of adolescents’ demographic background [including immigrant context (Delaruelle et al., 2021) and SES (Elgar et al., 2018)] with their mental health. Likewise, family support was shown to interact with social-environmental factors, such as teachers (Moore et al., 2018) and adolescents’ subculture affiliation (Bobakova et al., 2018).

Four unique studies measured the effects of family environment on adolescents’ health. One study investigated family environment as a protective factor against adolescents’ risk behaviours, injury, and psychosomatic symptoms longitudinally (Freeman et al., 2011). Likewise, another article tested the effect of home atmosphere, which was not mandatory in the HBSC survey (Simonsen et al., 2020). The last two studies combined types of family environmental psychosocial factors to probe the relationship between family and adolescents’ health outcomes (Simoes et al., 2008; Michaelson et al., 2016).

***Parenting behaviours (see the Supplementary Material “data extraction_T3_parenting”)***

Parenting behaviours (*N* = 100) were operationalised as parent-child communication (*N* = 62), parental monitoring (*N* = 32), parental emotional support (*N* = 17), parental promotion of autonomy (*N* = 8), school-related related support (*N* = 13), parent-child bonding (*N* = 4), parental rules (*N* = 7), and parenting style (*N* = 3).

Only one Spanish study investigated the trend in the relationship between parent-child communication and adolescents’ health. The other studies adopted a cross-sectional design. Only one paper discussed the mediation effect of parent-child communication. However, this study failed to find the obvious mediation in the association between family structure and adolescents’ alcohol use (Hoffmann, 2017). Six articles included the modifying effect of parent-child communication (Levin et al., 2012; Tomé et al., 2012; Resnick, 2014; Boniel-Nissim et al., 2015; Hong et al., 2020; Lackova Rebicova et al., 2020).

Three studies considered parental monitoring as a mediator. They indicated that parental monitoring mediates the association between demographic background and adolescents’ substance use (Wang et al., 2009; Vermeulen-Smit et al., 2012; Perasso et al., 2019). Four studies assessed the moderating role of parental monitoring. Two of them suggested that parental monitoring significantly moderated the gender (Jimenez-Iglesias et al., 2015) and age’s (Jimenez-Iglesias et al., 2013) effects on health outcomes. Additionally, parental monitoring might modify the social environment’s influence on adolescents’ health behaviours. One study noted parental monitoring decreased the risk of alcohol and cannabis use caused by adolescents’ perceived discrimination (Walsh et al., 2018). Among the studies that examined parental monitoring as an independent variable, only one article investigated the changes in parental monitoring’s effect on general life wellbeing across three-time waves. The results suggested a negligible influence of parental monitoring, and the trend was stable across waves (Jimenez-Iglesias et al., 2017).

The parental bonding instrument in the HBSC survey measures parental attachment behaviours toward children from two aspects, emotional support and autonomy promotion. Seventeen studies studied the relationship between parental emotional support and adolescents’ health, with the majority adopting substance use as outcomes (*N* = 8; Morgan and Haglund, 2009; Bobakova et al., 2012; Jiménez-Iglesias et al., 2012; Madkour et al., 2012; Jimenez-Iglesias et al., 2013; Zaborskis and Sirvyte, 2015; Sumskas and Zaborskis, 2017; Lee et al., 2020). Eight studies incorporated the sub-dimension “promotion of autonomy” of the parental bonding instrument to examine its link to adolescents’ health outcomes, including health behaviours (Morgan and Haglund, 2009; Jimenez-Iglesias et al., 2013; Zaborskis and Sirvyte, 2015), psychological health (Morgan et al., 2012; Klemera et al., 2017; Faltynkova et al., 2020), physical health aspect (McDyess, 2018), and global health reflection (Morgan and Haglund, 2009; Jimenez-Iglesias et al., 2015). A study demonstrated that parental emotional support and promotion of autonomy modified the correlation between gender and health-related quality of life (Jimenez-Iglesias et al., 2015). Moreover, four studies used parental bonding as a global variable and assessed its relationship with adolescents’ health-related behaviours (Ilona et al., 2012; Bobakova et al., 2013; Jovic et al., 2014; Cho and Norman, 2019).

Thirteen articles suggested the association between school-related parental support and adolescents’ health-related behaviours (Walsh et al., 2010; Vander Wal, 2012; De Clercq et al., 2014; Zaborskis and Sirvyte, 2015; Sumskas and Zaborskis, 2017; de Grado et al., 2018), physiological health (Matos et al., 2006; Sonmark and Modin, 2017), mental health (Matos et al., 2006; Danielsen et al., 2009; Plenty et al., 2014; Zaborskis et al., 2016; Madjar et al., 2018), and global health condition (Richter et al., 2012). A study suggested that parental support of school-related activities significantly buffered the negative influence of school demands on somatic health (Sonmark and Modin, 2017). Another study found that it did not moderate the relationship of school demands with emotional health and conduct problems (Plenty et al., 2014).

We synthesised seven articles that explored parental health-related rules’ effects on adolescents’ health behaviours. Only one article suggested the positive transfer effect of a specified parental health-related rule (Harakeh et al., 2012). It showed the influence of parental rules on alcohol drinking on adolescents’ alcohol/tobacco/cannabis usage and early sexual intercourse. The other studies focussed on certain health rules’ influence on the matched health behaviours. For instance, one article suggested that parental restriction of soft drinks decreased adolescents’ excessive consumption of soft drinks (Verzeletti et al., 2010b). Moreover, it was demonstrated that parental restrictions about alcohol use mediated the relationship between adolescents’ educational level and their excessive alcohol consumption (Vermeulen-Smit et al., 2012).

Three Lithuanian studies adopted a parenting style questionnaire in their national survey. They pointed out that parenting style correlated with adolescents’ substance use (Zaborskis and Sirvyte, 2015; Sumskas and Zaborskis, 2017) and their mental health (Zaborskis et al., 2016). The three Lithuanian studies also investigated the effects of the frequency of seeing parents and electronic communications with parents. The other two articles also included the independent variable of “seeing parents” (Levin and Kirby, 2012; Resnick, 2014).

***Parental health behaviours (see the Supplementary Material “data extraction_T4_ParentHealthBehave”)***

Several studies investigated parental health behaviours, specifically parental smoking behaviours (Rasmussen et al., 2005; Hublet et al., 2007; De Clercq et al., 2014), parental drinking (Kuntsche and Kuendig, 2006), parental general substance use (Bobakova et al., 2012), and parental physical activities (Wold et al., 1994; Bakalar et al., 2019). All parental health behaviours were seen as a direct predictor of their adolescent children’s health behaviours. For example, parental smoking behaviour was a predictor of adolescents’ tobacco consumption.

***Family activities (see the Supplementary Material “data extraction_T5_FamilyAcitivity”)***

In terms of the studies on the effects of family activities on adolescents’ health outcomes, the majority investigated the effects of diverse family activities as predictors (*N* = 13). Two studies also explored the interaction effect of family activity with adolescents’ gender and age (Jimenez-Iglesias et al., 2013, 2015).

Besides, ten studies focussed on family meal activity. Four of them specified weight concerns (Kelly et al., 2016), weight control behaviours (Tur-Sinai et al., 2020), and food habits (Verzeletti et al., 2010a,b) as outcomes, which were highly correlated with the actual BMI and body image. Additionally, one article focussed on the relationship between family meal routines and adolescents’ body image (Ramseyer Winter et al., 2019). However, the effect of family meals on adolescents’ substance use was inconsistent. Family dinner correlated with adolescents’ use of alcohol, tobacco, and cannabis in one study (Levin et al., 2012a), while another study suggested family dinner was neither directly linked to adolescents’ alcohol consumption nor modified the relationship between parental control and alcohol use (Perasso et al., n.d.). Family dinner routine was also correlated with adolescents’ toothbrushing (Levin and Currie, 2010).

Only two studies focussed on family physical activity. Family physical activity was correlated with adolescents’ physical activity itself (Bakalar et al., 2019). The other study based on the HBSC data from 37 European and North American countries suggested that physical custody arrangement after parental divorce promoted adolescents’ general life satisfaction (Steinbach et al., 2021).

***Siblings (see the Supplementary Material “data extraction_T6_sibling”)***

Four articles indicated that the number of siblings in a family’s influences adolescents’ food habits (Rouche et al., 2019; Fismen et al., 2020), oral health behaviour (Levin and Currie, 2010), and reproductive health (Steppan et al., 2019). Moreover, three studies evaluated the relationship between communication with siblings and adolescents’ health outcomes. However, only one study found that close communication with siblings was a significant protective factor of adolescents’ regular breakfast (Levin and Kirby, 2012).

***Mediation and moderation analyses (see the Supplementary Material “data extraction_MedMod”)***

Compared to the moderation analysis, fewer studies using the HBSC data conducted mediation analyses. Six studies investigated the mediating role of parental monitoring (Wang et al., 2009; Vermeulen-Smit et al., 2012), communication with parents (Hoffmann, 2017), parental attachment (Cho and Norman, 2019), parental health-related rules (Vermeulen-Smit et al., 2012), family control (Perasso et al., n.d.), and family members with whom adolescents live (Steppan et al., 2019).

Sixty-nine studies examined the moderating role of family-related factors, with 22 studies investigating the inequality in diverse health outcomes of adolescents, such as substance use, food habit, and physical and psychological health. These studies provided evidence that SES moderated the trend of adolescents’ health inequality over time, indicating that the assessed health outcomes differed across different SES classes. Likewise, two studies indicated that family structures influenced adolescents’ breakfast consumption frequency (Levin et al., 2012b; Sumskas et al., 2012). Several studies tested whether the family environmental contributors interacted with individual-level variables, such as gender (Ahlborg et al., 2017) and individual health risk behaviours (Boyce et al., 2008). Within-family interactions were also examined. For instance, a study suggested that the low satisfaction with family increased the negative effect of parental unemployment on adolescent girls’ mental health (Frasquilho et al., 2017). Family environment modified the effects of adolescents’ direct social context, such as school (Freeman et al., 2011) and peer relationships (Tomé et al., 2012; García-Moya et al., 2017; Hong et al., 2020). A study also found that family-level interacted with country-level context as well. Parental and country unemployment worsened the adolescents’ general life satisfaction (Johansson et al., 2019).

A Slovak study simultaneously investigated the mediator and moderator of family affluence and structure. However, the results revealed that family affluence and family structure did not mediate or moderate the relationship between adolescents’ learning disabilities and their health risk-taking behaviours (Palfiova et al., 2017).

### Hypothesis-Driven and Data-Driven Studies

Hypothesis and data-driven analysis are two useful approaches in the public health field. The first method is based on prior information. The technique helps us examine a certain assumption generated from previous findings and theories to explain the exact phenomenon (Previdelli et al., 2016). The data-driven approach considers the quantitative relationships between variables without prior hypotheses. It provides potential powerful predictive models. A data-driven approach is advanced when there is no existing theory, or the associations of variables are too complicated to be observed (Cox et al., 2020). HBSC provides abundant information on adolescents’ environmental context. Therefore, the data-driven method may reveal underlying influences of environments on adolescents’ health-related outcomes not supported by previous theories. Especially, it potentially demonstrates the interactions between the environmental contributors. However, only five studies used data-driven approaches.

One of the articles used a Bayesian network to investigate the protective effects of family, school, and peer supports on low life satisfaction and health complaints. The results suggested that family and school support were stronger protective factors compared to peer support (Borraccino et al., 2020). Three studies adopted decision tree models to determine the main contributors to adolescents’ mental health. They all pointed out the importance of the family’s psychosocial functions and good parent-child relationships (Morgan et al., 2012; Garcia-Moya et al., 2013; Moreno-Maldonado et al., 2020a). One study employed a data-driven approach to investigate the health risk behavioural outcomes rather than the potential influencers that may affect health outcomes (dos Santos et al., 2020). It adopted the cluster analysis to identify individuals with similar health risk-taking behaviours.

## Discussion

Our discussion focuses on interpreting the findings to answer the main question of this study: What family environmental factors were included in the previous adolescents’ health-related studies based on the HBSC database? What are the research gaps? Finally, we pointed out the limitations of this scoping review.

### Family Environmental Factors

Unlike the systematic review, which aims to synthesise related evidence from previous studies, scoping review addresses the extent of available evidence. Usually, researchers categorise the findings into groups in various ways (Aromataris and Munn, 2020). The current research aims to classify the studies that examined the effects of family-related factors on adolescents’ health outcomes based on the HBSC database.

Family environmental factors can be categorised into six categories: family demographic backgrounds, general family’s psychosocial functions, parenting behaviours, parental health behaviours, family activities, and siblings.

Socioeconomic status is one of the most important demographic background variables. However, as SES is a complex concept, previous scholars have assessed this variable from diverse perspectives, such as family affluence, parental occupational social class, parental or individual educational level, and subjective feelings of family wealth. Some studies calculated family relative SES based on family affluence, such as the Yitzhaki index. The second notable issue is that health inequality across different SES classes has been considered since 2007. Health inequality may vary over time, and the trend in change may differ across countries; therefore, it is necessary to investigate health inequality cross-culturally to develop targetted interventions (Holstein et al., 2020, 2021). Compared to the studies focussing on family SES, the number of studies on the family structure is relatively small (see the Supplementary Materials “data extraction_T1_back”). Sixty-seven studies investigated the effect of family structure on adolescents’ health-related outcomes. In Europe, divorce rates and the number of single-parent households are growing (Amato, 2014; Eurostat, 2015). A similar pattern is also found in North America (McMillan et al., 2015) and China (Lijuan and Qi, 2016). Literature suggests that the single-parent and reconstituted family structure are potential risk factors for adolescents’ cognitive, emotional, and physical development (Ram and Hou, 2003; Langoy et al., 2019). Concerns about family structure are also reflected in the number of related studies based on the HBSC database. From 2001 to 2010, fifteen studies investigated the relationship between family structure and adolescents’ health outcomes. From 2011 to 2020, the number of relevant studies rose to 52. Besides, the number of adolescents from immigrant backgrounds has been gradually increasing, which leads to a growing interest in their health. According to the HBSC database, around 5% of adolescents are the first and 14% are second-generation immigrant adolescents. Since 2006, HBSC-based studies have also been exploring immigrant status influences.

We found family general psychosocial function is the second important category. It is consistent with the Olson Annular Mode Theory, which suggests that family has three psychosocial functions, family intimacy, family adaptability, and family communications (Dai and Wang, 2015). Family intimacy emphasises the relationship between family members. HBSC includes items measuring adolescents’ satisfaction with family relationships. HBSC program contains mandatory family communication measure as a crucial factor underlying family dynamics. Family adaptability, which refers to family rules or roles of a family member in adapting to the external environment, is not assessed directly in the HBSC program. Yet, HBSC contains a section assessing parental rules toward adolescents’ behaviours. Ample studies have suggested that family psychosocial functions are highly correlated with adolescents’ health behaviours, physical health, and psychological health (Berge et al., 2013; Halliday et al., 2014; Ferro and Boyle, 2015).

Parents are critical role models for adolescents. Their parenting behaviours can be an independent factor influencing adolescents’ health outcomes. The finding aligns with the earlier theory that parenting style and attitudes significantly affect youth development. For instance, permissive parenting is associated with adolescents substance dependency (Bogenschneider et al., 1998), and parental over-involvement is another potential risk factor of adolescents’ addiction disorder (Xiuqin et al., 2010). Additionally, a democratic parent-child relationship improves physical health conditions (Berge et al., 2010).

Parents’ health behaviour is an additional significant predictor of adolescents’ health outcomes, especially their health behaviours. Social norms, usually reflected in the health attitude or behaviours within the social environment, influence adolescents’ health-protective or risk behaviours. Thus, parental health behaviours affect adolescents’ attitudes toward and cognition of health-related behaviours, such as substance use (Rasmussen et al., 2005; Kuntsche and Kuendig, 2006).

The fifth category of family environmental factors associated with adolescents’ health is a family activity. Even though family activity does not provide psychosocial support directly, it boosts the sense of family belonging, which protects adolescents from developing health risk behaviours to some extent (Morgan and Haglund, 2009; Brooks et al., 2012). Adolescent-to-family fit theory suggests that engagement in shared family activities forms a reciprocal relationship between adolescents and the family, satisfying adolescents’ emotional needs and eventually reducing their health risk-taking behaviours, such as substance use (Mccubbin et al., 1985). A piece of empirical evidence not based on HBSC data suggested that family activities benefit adolescents’ mental health (Compañ et al., 2002). Moreover, an international survey study noted that family activities are consistently associated with adolescents’ wellbeing across nations (Lee and Yoo, 2015).

Considering family dynamics, siblings have not been given sufficient attention in research. The synthesised evidence provides inconsistent evidence of the positive influence of the relationship with siblings on adolescents’ health behaviours.

### Research Gaps

The current study addressed several prominent research gaps.

First, the persistent health inequality trend is not stable across countries. However, only six European countries conducted the related research. Among these studies (*N* = 21), Denmark studies occupy the biggest portion (*N* = 11). The relevant studies in other European countries are inadequate, and to our knowledge, no such study has been conducted in North America.

Second, as suggested, youth are living increasingly in types of family structures. It is valuable to examine the changes in the health inequality caused by family structures among adolescents. However, only two studies probed the issue.

Third, the effect of immigration status on health is controversial and worth examining, especially since the studies conducted in two North American countries have ignored this effect. According to the 2016 report, the newcomers under 24 years old accounted for 1/3 of the Canadian population (Chui and Flanders, 2017). The proportion of immigrant children under 18 years old was 27% in the United States (Child Trends, n.d.). Future studies need to pay attention to immigrant adolescents’ health-related outcomes in the North American region. Immigration, which has become a vital issue globally, challenges the health system and people’s wellbeing (Trost et al., 2018). Unfortunately, no study has explored changes in immigrant adolescents’ health outcomes over time using the HBSC database, even though the database provides abundant relevant information.

Fourth, siblings provide social interaction experiences, serving as a psychosocial resource to benefit adolescents’ development (Feinberg et al., 2013). A meta-analysis suggested that more sibling warmth is associated with less internalising and externalising problems (Buist et al., 2013). However, on the other hand, siblings’ antisocial behaviours, such as conspiring against parental authority and delinquent behaviours, provide each other with models of deviant behaviours (McHale et al., 2012). From this perspective, sibling relationships may bring some negative impacts. Thus, the effect of the relationship with siblings remains unclear and needs more deep investigations.

Fifth, a limited number of studies have conducted mediation and moderation analyses, especially the mediation models (*N* = 6). Even though some research studies investigated family interactions utilising the moderation analysis, no mediation model explored the mechanisms underlying dynamic family interactions in our synthesised research. For instance, the pathway from parent-child interactions, family psychosocial features, and parent-sibling relationships to adolescents’ health outcomes remains unclear.

Finally, as discussed, HBSC provides contextual information to help researchers identify social determinants of adolescents’ health outcomes. Compared to the hypothesis-oriented method, data-driven analysis is a better solution to handle mass variables. However, only four studies explored the potential family factors related to adolescents’ health, and three of them adopted decision tree models to select possible family-related predictors without causal inference.

### Limitations

This scoping review work had some limitations. First, unlike a systematic review, scoping review itself is not interested in a precise question, for instance in this study case, a question like “the effect size of a family-related factor on adolescents’ health behaviour/wellbeing.” Instead, scoping review leads to a broader and less defined search and screening, and it addresses the extent of previous evidence and the identify the existing research gap. Scoping review does not review a precise set of outcomes evidence (Aromataris and Munn, 2020). Thus, in our scoping review, we could not answer the questions about specific evidence for instance, we did not investigate if the effect of family factors was moderated by other variables through multivariate analysis.

Second, it was worth noticing the constantly changing questionnaires in the HBSC project crossing years and countries, which meant the evidence on a specific question was limited by time and region (Currie et al., 2010, 2014; Inchley et al., 2018). For instance, immigrant information related questions (“Migration” section in HBSC) have been only mandatorily included since 2013, which meant not all HBSC members collected the migrant information before 2013 and the regional evidence in adolescents’ immigrant background was not sufficient. Likewise, in the “family culture” section, numbers of siblings and communication with siblings have no longer been compulsory questions since 2009. Also, the family communication related items (e.g., “when I speak someone listens to what I say”) were only mandatorily required in the HBSC2013/2014. Regarding the social inequality, HBSC2017/2018 did not require to code parental occupational social status anymore.

## Conclusion

As one of the direct contexts with which adolescents interact, the family environment serves a significant psychosocial function in adolescents’ physical and psychological development. Other elements in the family environment, such as demographic characteristics, parents, siblings, and organised sharing activities, also influence adolescent health. However, the longitudinal examinations of the association between demographic background and health inequality are limited. Additionally, the effects of family environmental contributors on adolescents’ health outcomes vary across different contexts. It is necessary to conduct replications critically in the future. Additionally, insufficient studies have focussed on path analysis to explore the mechanism of the interactions within the family environment. Last, as HBSC provides abundant social background information, further studies should adopt more data-driven analysis to identify critical contextual factors related to adolescents’ health.

## Author Contributions

YH made substantial contributions to the data analysis and interpretation, conception and design of the manuscript, drafting the manuscript, and revising it critically. MP, JL, AR, and PM made substantial contributions to the data analysis and interpretation and drafting and revising of the manuscript. YH and MP collected the data and contributed to the data analysis. YH and JL made substantial contributions to the conception and design of the study, critically revised the manuscript, and approved the final version to be published. All authors certified that they have participated sufficiently in the work.

## Conflict of Interest

The authors declare that the research was conducted in the absence of any commercial or financial relationships that could be construed as a potential conflict of interest.

## Publisher’s Note

All claims expressed in this article are solely those of the authors and do not necessarily represent those of their affiliated organizations, or those of the publisher, the editors and the reviewers. Any product that may be evaluated in this article, or claim that may be made by its manufacturer, is not guaranteed or endorsed by the publisher.
