# Supplementary material for: Family Related Variables’ Influences on Adolescents’ Health Based on Health Behaviour in School-Aged Children Database, an AI-Assisted Scoping Review, and Narrative Synthesis
Source: Front Psychol. 2022 Aug 10;13:871795. doi: 10.3389/fpsyg.2022.871795 (PMC9400839; doi:10.3389/fpsyg.2022.871795)
Supplement: Supplementary file 1 [file Data_Sheet_1.docx]

**Instruction of supplementary materials**

**Brief summary of this document:** this document was an instruction about Excel files. Each Excel file included a special batch of extracted data. The following noted the content of each Excel file.

**1. "data extraction_ALL":**

Information of all the articles which were used for analysis.

**2. "data extraction_MedMod":**

The extracted information about articles that adopted statistical mediation or moderation model(s). There were three worksheets in the file. The 1st worksheet included articles that adopted mediation analysis. The 2nd sheet included articles that adopted moderation analysis. The final one included articles adopting both mediation and moderation analysis.

**3. "data extraction_T1_back":**

This file focused on the first theme of family-related variables, demographic background. All the articles in this file could extract demographic background related factors. The first three worksheets included articles that can extract a certain type of demographic data, for instance, SES. The 4th worksheet was a synthesis of all the articles including demographic data.

**4. "data extraction_T1_HouseCom":**

All the articles in this file can extract the household composition data. As described in the paper, the household composition was one of the measurements of family structure, which belonged to the theme "demographic background".

**5. "data extraction_T1_SES_Edu":**

This file included articles adopting parental or personal education level to measure SES, which was one of the factors belonging to the main theme "demographic background".

**6. "data extraction_T1_SES_FAS":**

All the articles in this file can extract SES factor measured by Family Affluence Scale (FAS).

**7. "data extraction_T1_SES_OSC":**

This file was a synthesis of articles adopting parental occupational social class to measure SES.

**8. "data extraction_T2_GeneralPsySocio":**

This file was a synthesis of articles that can extract the second main theme, the general family's psychosocial functions. This file included five worksheets, and articles in each worksheet can extract a certain type of family general psychosocial factor, such as family support and family communications. The final worksheet was the synthesis of all articles that can extract the second theme.

**9. "data extraction_T3_parenting":**

All the articles in this file measured the relationship between parenting behaviours (the third theme) and adolescents' health outcomes. Each worksheet in the file included a certain type of parenting behaviours, for example, parent-child communications and parental monitoring. The final worksheet was the synthesis of all articles that can extract the third theme.

**10. "data extraction_T4_ParentHealthBehav":**

This file synthesized the fourth theme of family variables that may influence adolescents' health outcomes. The theme included four types of parental health behaviours: parental smoking (1st worksheet), parental substance use (2nd worksheet), parental drinking (3rd worksheet), and parental physical activities (4th worksheet). The 5th worksheet was the synthesis of all articles that can extract the fourth theme.

**11. "data extraction_T5_FamilyActivity":**

Articles synthesized in this file were focusing on the fifth theme of family factors: family activity. Worksheets in the file noted the articles that can extract global family activities, family meals, and family physical activity. The final worksheet was the synthesis of all articles that can extract the fifth theme.

**12. "data extraction_T6_sibling":**

Articles synthesized in this file were focusing on the sixth theme of family factors: siblings. The final sheet was the whole synthesis. The 1st sheet noted the articles that can extract sibling(s) communication data. The 2nd worksheet addressed the number of siblings.
